# Association between heart rate variability and ECG changes in on-duty prehospital physicians

**DOI:** 10.3389/fphys.2025.1617377

**Published:** 2025-07-23

**Authors:** Mathias Maleczek, Karl Schebesta, Thomas Hamp, Balthasar Laussner, Thomas Pezawas, Mario Krammel, Bernhard Roessler

**Affiliations:** ^1^ Medical Simulation and Emergency Management Research Group, Medical University of Vienna, Department of Anaesthesia, Intensive Care Medicine and Pain Medicine, Clinical Division of General Anaesthesia and Intensive Care Medicine, Vienna, Austria; ^2^ Academic Simulation Center Vienna, Vienna, Austria; ^3^ Department of Anaesthesia and Intensive Care Medicine, Orthopaedic Hospital Vienna – Speising, Vienna, Austria; ^4^ Emergency Medical Service Vienna, Vienna, Austria; ^5^ Department of Medicine II, Division of Cardiology, Medical University of Vienna, Vienna, Austria; ^6^ PULS – Austrian Cardiac Arrest Awareness Association, Vienna, Austria

**Keywords:** HRV, stress, emergency, critical care, prehospital, ECG, occupational health

## Abstract

**Background/objectives:**

Prehospital emergency physicians work in physically and psychologically stressful environments. During their shifts, changes in electrocardiogram (ECG) attributable to stress have been described previously. Alterations in heart rate variability (HRV) as well as in ST-T segments have been reported. Nevertheless, the association between those two parameters still remains unclear in this setting.

**Methods:**

A retrospective analysis of data collected in a previous prospective trial was conducted. The primary objective was the association of HRV metrics with the risk of ST-T abnormalities during 5-min intervals and on a mission basis. Therefore, the root mean square of successive differences (RMSSD) and standard deviation of normal-to-normal (SDNN) intervals were analysed. Additionally, variations in HRV during different phases of a mission were investigated.

**Results:**

Data of 20 physicians was analysed. SDNN was positively associated with ST-T abnormalities both on a 5 min basis (OR: 1.04, 95%CI: 1.03-1.04) and a mission basis while RMSSD was negatively associated with ST-T abnormalities evaluated per mission (OR: 0.73, 95%CI: 0.57-0.93). pNN50 was not associated with ST-T abnormalities. During patient care and patient transport HRV was significantly lower than during alarm and en-route of a rescue mission.

**Conclusion:**

No reliable correlation between HRV values and the occurrence of ST-T segment changes during missions in prehospital emergency physicians were found. Therefore, it is questionable whether HRV alone is sufficient to detect ischemia-like changes during stressful events.

## 1 Introduction

Stress induces adaptive responses in the cardiovascular system, which can manifest as alterations in the electrocardiogram (ECG). This was previously shown in multiple studies including one recent trial by the authors (Luurila et al., 1994; Doorey et al., 2011; Herm et al., 2017; Maleczek et al., 2022). In the latter study, 70% of 20 previously health prehospital emergency physicians had ST-T segment changes while on duty. Although ST-segment deviations—the primary outcome—were documented twice, the majority of changes consisted of T-wave inversions (n = 124). No significant correlation was found between ECG alterations and subjective workload as measured by the NASA Task Load Index. However, alarms occurring during sleep were associated with a significantly higher incidence of ST-T segment changes.

Stress–especially experienced in a workplace environment - can lead to a multitude of physiological and psychological effects, with the most significant including an increased risk of cardiac events, burnout, and fatigue (Ho et al., 2010; Guasch and Mont, 2017). Both mental and physical stress leads to ST-T segment changes which can be seen as sign of cardiac ischaemia (Byrne et al., 2023). This is probably a contributing factor to emergency-physicians having a shorter life expectancy compared to the general population (Brayne et al., 2021).

Heart rate variability (HRV) has been widely studied as a physiological indicator of psychological stress (Hernando et al., 2018; Gupta et al., 2022; Himariotis et al., 2022). Recent literature has consistently demonstrated associations between heart rate variability, psychological stress, and clinical outcomes. In particular, reduced HRV—most notably reductions in RMSSD—has been linked to elevated stress levels. However, HRV is influenced by a variety of factors beyond psychological stress, including environmental conditions, lifestyle habits, and genetic predispositions (Tiwari et al., 2021; Immanuel et al., 2023) In a general population mortality could be predicted using HRV (Jarczok et al., 2022).

It is well established that the activity of the autonomic nervous system—particularly the parasympathetic branch—strongly influences heart rate variability (HRV), which is derived from the temporal variations between successive R-R intervals in the electrocardiogram (ECG). A wide array of HRV metrics have been described in the literature, encompassing time-domain, frequency-domain, and non-linear measures (Shaffer and Ginsberg, 2017; Thomas et al., 2019; Pham et al., 2021) Whilst some of these values are validated for long term (24 h) observations, one of the most robust metrics in short term observations is the root mean square of successive differences (RMSSD): It mainly reflects vagal activity and the body’s ability to recover (Shaffer and Ginsberg, 2017; Kim et al., 2018; Thomas et al., 2019). In contrast, the standard deviation of normal-to-normal intervals (SDNN) is more broadly influenced by both sympathetic and parasympathetic inputs and is widely used as a general marker of autonomic balance. SDNN has been extensively studied as a prognostic indicator, particularly in cardiology, where reduced values have been associated with increased risk of adverse outcomes, including cardiac mortality post-myocardial infarction (Kleiger et al., 1987; Shaffer and Ginsberg, 2017). While SDNN captures total variability influenced by both sympathetic and parasympathetic activity, RMSSD is more specifically associated with short-term, high-frequency variations primarily mediated by parasympathetic tone. As a result, their correlation may vary depending on the recording duration, physiological state, and context, with stronger correlations typically observed in resting conditions and short-term measurements. RMSSD is more selectively modulated by parasympathetic activity than SDNN, making it particularly suitable for detecting rapid shifts in vagal tone. Both metrics have shown utility across various domains: in clinical medicine (e.g., predicting sudden unexpected death in epilepsy), psychiatry (e.g., assessing autonomic dysregulation in post-traumatic stress disorder), and sports science (e.g., monitoring training load and recovery) (DeGiorgio et al., 2010; Schmitt et al., 2015; Schneider and Schwerdtfeger, 2020) Some authors of technical literature suggested the use of the easily accessible HRV as potential surrogate for ST-T segment changes (Udhayakumar et al., 2019).

All these factors lead to HRV being a strong biomarker of stress and therefore occupational health (Sammito et al., 2024). HRV can even be recorded with a smart watch, although methodical issues were currently published: It has been shown that photoplethysmography derived HRV shows a non-uniform measurement error and therefore cannot replace ECG derived HRV (Hernando et al., 2018; Dewig et al., 2024). Both metrics have actually been used in prehospital medicine: Different trials report the usage of HRV generated by different recording devices but all without analysis of a 12-lead ECG especially without reporting ST-T segments (Petrowski et al., 2019; Schöniger et al., 2020a; Schöniger et al., 2020b). As described, the occurrence of ST-T segment changes is much more associated with cardiac risk than HRV (Lakusic et al., 2015; Deng et al., 2020; Hakim et al., 2020; Liu et al., 2020). Therefore, it is of great interest if HRV can predict ST-T segment changes. This would simplify future trials as no complex 12-lead holter ECGs would be necessary and the occurrence of ST-T segment changes could be assumed.

Consequently, association between HRV metrics and ST-T segment changes in healthy prehospital emergency physician was investigated using a dataset collected during a previously conducted prospective trial (Maleczek et al., 2022). The primary aim of this study was to investigate associations between HRV and ST-T segment changes The comparison of SDNN and RMSSD performance as surrogate for ST-T segment changes was designated as a secondary outcome.

## 2 Materials and methods

This study is a secondary analysis of a previous trial by the authors (Maleczek et al., 2022). The local ethics committee approved this analysis (reference number: 1,038/2023). The original trial was registered at ClinicalTrials.gov (NCT04003883). Shifts were included in the original trial between 2019 and 11–15 and 2021-03-27, participants prospectively gave written consent to analysis of both ST-T segment changes and changes in HRV. Analysis was done using a 12-lead Holter ECG (FD12+, Schiller AG, Switzerland) during day and night shifts.

Previously healthy participants were recruited from the regular staff doing shifts at the Medical University of Vienna’s prehospital emergency physician’s car. This vehicle is staffed with a paramedic and a prehospital emergency physician with at least 3 years of training in either Internal medicine or Anaesthesia. Participation was strictly voluntary without any compensation. All participants analysed in the original trial were used for this analysis.

After inclusion, participants were thoroughly tested for cardiovascular disease using resting ECG, 24-h ECG, blood test, echocardiography and ergometry. Participants with abnormal findings in baseline investigations were excluded from the data analysis.

For the final dataset a baseline-24 h ECG and one day- and/or nightshift ECGs (8–16 h) were obtained. A detailed description of data acquisition is provided in the previous publication (Maleczek et al., 2022). For this secondary analysis, ECG data of all originally included 20 participants was investigated retrospectively. Data was accessed on the 2023–02–15 for this retrospective analysis with no way of identifying probands from the used data.

Primary outcome was the association of different HRV metrics with immediate ST-T abnormalities. Secondary outcomes were associations between mean HRV values during a call and the occurrence of ST-T abnormalities. Furthermore, descriptive statistics of HRV values during the shift were calculated.

### 2.1 ECG processing

HRV was calculated with the medilog Darwin software (V2 2.* - Schiller AG, Switzerland, 2017). RMSSD, SDNN and pNN50 were calculated automatically with 5-min intervals. Further data analysis was done using Python 3.8 using pandas, numpy, seaborn, matplotlib and scipy (McKinney, 2010; Harris et al., 2020; Virtanen et al., 2020; Waskom, 2021).

ST-T segment changes were manually annotated by one of the authors (MM) under the supervision of a senior cardiologist (TP) in the original trial. T-wave inversions were defined as new-onset negativity of the T-wave of any amplitude, persisting for at least two consecutive beats. ST-T segment changes were categorized based on their duration (≤30 s vs >30 s) and amplitude (≤0.1 mV vs >0.1 mV). For this trial all ST-T abnormalities were used regardless of type and duration. As HRV metrics RMSSD, SDNN, and pNN50 were used as calculated by the medilog software.

### 2.2 Statistical analysis

To examine the association between HRV-metrics and ST-T abnormalities, a mixed-effects logistic model was applied, incorporating physician-level variability as a random intercept. Fixed effects included the HRV parameters SDNN, RMSSD, and pNN50. The primary analysis was conducted on 5-min interval data derived from continuous ECG recordings.

For this secondary analysis, data were aggregated at the mission level by calculating mean values of HRV metrics per mission, and ST-T abnormalities were coded as a binary outcome. A comparable mixed-effects modelling approach was employed. All statistical analyses were performed using R (lme4 package) and Python (scipy package) (Van Rossum and Drake Jr, 1995; R Core Team, 2017), and regression coefficients with corresponding 95% confidence intervals were visualized using forest plots.

Two sensitivity analyses were conducted to test the influence of sex and heart rate on the stability of the shown results. Finally, HRV metrics between mission phases were compared using a Friedman test.

## 3 Results

Twenty emergency physicians were included in this secondary analysis. Mean age of the participants was 39 (standard deviation (SD): 4) years, 40% were female. Except of for four physicians who only did one shift, all others did one night and 1 day shift, resulting in 36 recorded (18 days and 18 night) shifts with a total of 208 rescue missions. In the data set 124 ST-T abnormalities (98.4% T-wave inversions) were found (Maleczek et al., 2022).

Large differences in between mean RMSSD, SDNN and pNN50 values were found: The overall mean RMSSD was 30.5 m (SD: 12.8) whereas the overall mean SDNN was 77.4 m (SD: 23.3). Mean pNN50 was 11 (SD: 9.8). Details are described in Table 1.

**TABLE 1:** Details of heart rate variability parameters during different time periods are shown.

Phase	n	RMSSD [ms] mean (SD)	SDNN [ms] mean (SD)	Heartrate [/min] mean (SD)
Overall	202	30.5 (12.8)	77.4 (23.3)	80.4 (13.0)
Day shift	123	31.3 (14.3)	75.7 (23.5)	80.8 (12.6)
Night shift	79	29.2 (10.1)	80.0 (22.7)	79.4 (14.1)
Alarm	184	31.7 (12.9)	89.2 (32.3)	78.6 (12.5)
En-route	149	31.3 (12.2)	81.7 (30.0)	78.6 (12.5)
Pt. care	57	25.7 (11.8)	67.0 (22.0)	90.9 (13.6)
Pt. transport	54	26.1 (12.0)	63.4 (21.9)	86.5 (12.0)
ST-T change	51	31.0 (12.2)	82.4 (26.1)	80.8 (13.3)

n: number, Alarm: Time of alarm, En-route: Driving to the patient, Pt. care, Time caring for the patient until transport to hospital. Pt. transport, Time in the ambulance with the patient on the way to the hospital, ST-T, change; HRV, metrics during missions with at least one ST-T, segment changes.

**FIGURE 1:**
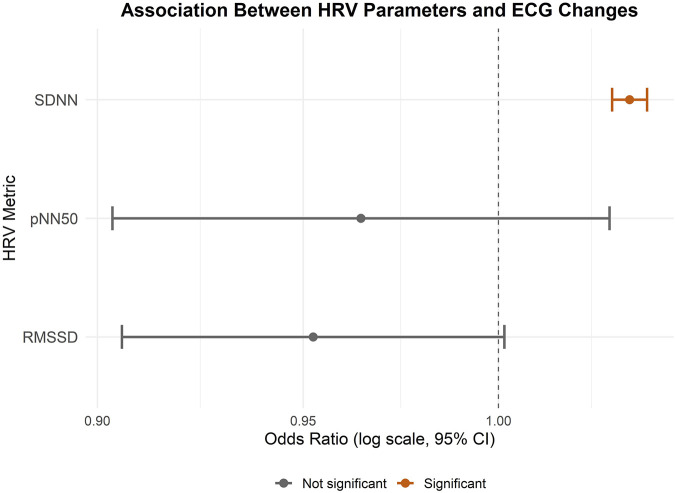
Forest Plot showing the associations between HRV parameters and ST-T abnormalities. An odds ratio (OR) greater than one indicates an increased likelihood of ECG changes associated with the predictor variable, whereas an OR less than one suggests a decreased likelihood.

In the conducted mixed-effects logistic regression model testing associations between the model was based on 10,626 observations across 20 emergency physicians (mean number of observations per person: 531.3), and the analysis converged successfully. The results revealed a statistically significant positive association between SDNN and immediate ST-T abnormalities (OR = 1.04, 95% confidence interval (CI) = 1.03-1.04), suggesting that higher SDNN values were associated with an increased probability of ST-T abnormalities. Neither RMSSD (OR = 0.95, CI = 0.91-1.00) nor pNN50 (OR = 0.96, CI = 0.90-1.03) showed a significant association with ST-T abnormalities. Model performance showed an AIC of 1,449.1, BIC of 1,485.5, and log-likelihood of −719.6. The random intercept variance was 4.57 (SD = 2.138), indicating substantial between-subject variability (Figure 1).

**FIGURE 2:**
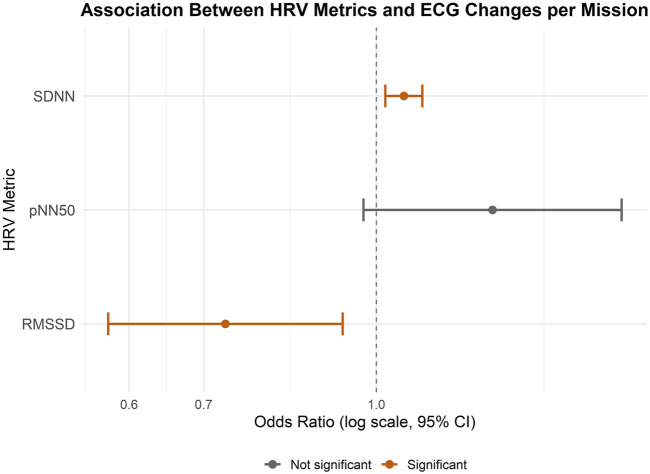
Forest Plot showing the associations between HRV parameters and ST-T abnormalities on mission level. An odds ratio (OR) greater than one indicates an increased likelihood of ECG changes associated with the predictor variable, whereas an OR less than one suggests a decreased likelihood.

In the mission-level analysis, the association between HRV parameters and the presence of any ECG change during this mission was assessed using logistic regression. The model included three predictors: RMSSD, SDNN, and pNN50, each representing overall mean values per mission. Among the predictors, SDNN (OR = 1.06, CI = 1.02-1.10) and RMSSD (OR = 0.73, CI = 0.57-0.93) were significantly associated with ST-T abnormalities suggesting that higher SDNN values but lower RMSSD values were associated with increased odds of observing ECG abnormalities. pNN50 was not significantly associated with the outcome (OR = 1.27, CI = 0.97-1.66). This model showed an AIC of 204.6, BIC of 221.1, and log-likelihood of −97.3 indicating a better balance of fit and simplicity. The variance of the random intercept was 3.39 (SD = 1.84), indicating notable between-subject variability (Figure 2).

To test for the influence of sex on the association of HRV metrics on ST-T abnormalities, a sensitivity analysis was done adding sex as a fixed effect to the including all ST-T abnormalities: In this extended model, sex was added as a fixed effect alongside the original HRV predictors (SDNN, RMSSD, pNN50). Notably, female sex was significantly associated with increased odds of ST-T abnormalities (OR = 16.12, 95% CI: 2.99–87.01, *p* = 0.001), even after adjusting for HRV parameters. The effect of SDNN remained robust (OR = 1.035, 95% CI: 1.031–1.040, *p* < 0.001), while RMSSD continued to show a trend toward a protective effect (OR = 0.951, 95% CI: 0.904–1.001, *p* = 0.056).

In another sensitivity analysis, heart rate was included into the generalized linear mixed model. The model demonstrated good fit (AIC = 1,404.9). As before, higher SDNN values were positively associated with the likelihood of ST-T segment changes (OR = 1.033, 95% CI: 1.028–1.038, *p* < 0.001), while pNN50 showed a modest negative association (OR = 0.937, 95% CI: 0.878–0.999, *p* = 0.047), suggesting that lower parasympathetic activity may relate to the occurrence of ST-T abnormalities. Mean heart rate also emerged as a significant positive predictor (OR = 1.055, 95% CI: 1.040–1.071, *p* < 0.001). RMSSD was not significantly associated with ST-T abnormalities in this model.

**FIGURE 3:**
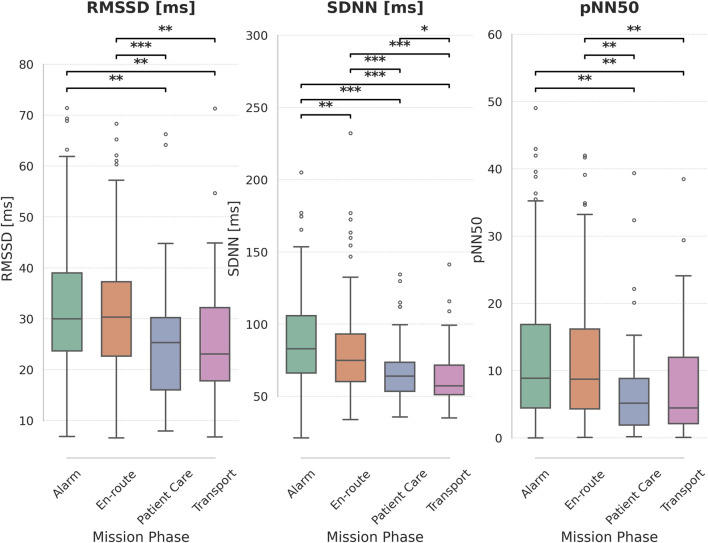
Heart Rate Variability (HRV) metrics across mission phases. Boxplots display the distribution of RMSSD [ms], SDNN [ms], and pNN50 for each mission phase: Alarm, En-route, Patient Care, and Transport. Significant pairwise differences between mission phases are indicated by asterisks above the plots (*: *p* < 0.05, **: p < 0.01, ***: p < 0.001), based on paired t-tests with Holm-Bonferroni correction for multiple comparisons.

Comparing HRV metrics between different phases of missions show significant differences for all three metrics (Friedman test, p < 0.001 for all three). Details can be found in Figure 3.

## 4 Discussion

In this study, a possible association between HRV and the occurrence of ST-T segment changes including T-wave inversions was investigated. Finding such an association has the potential to simplify studies researching the cardiac influence of stress–especially in physically demanding areas where wearing an ECG-holter can even be dangerous.

SDNN was found to be predictive of ST-T segment changes both in 5-min intervals and on a mission-level basis. Notably, higher SDNN values were associated with an increased likelihood of ST-T abnormalities—an unexpected direction of association. This is in contrast with existing literature, where lower SDNN values have typically been associated with increased stress (Schöniger et al., 2020a; Schöniger et al., 2020b). The reason for this interesting finding remains unclear as it contradicts common knowledge of HRV metrics associated with outcomes (Sammito et al., 2024). Even after correcting for heart rate, this positive association persisted. This is in line with previous data in air rescue physicians, where SDNN was lowest on the days off (Schöniger et al., 2020b).

Furthermore, RMSSD showed no association with ST-T abnormalities in the short-term (5-min interval) analysis but demonstrated a significant association at the mission level. In this context, lower RMSSD values corresponded with a higher risk of ST-T abnormalities, aligning with established expectations. This pattern may reflect the distinct physiological mechanisms involved: HRV captures autonomic nervous system activity, whereas ST-T segment changes are generally considered markers of transient myocardial perfusion mismatch (Park et al., 2017; Shaffer and Ginsberg, 2017; Kim et al., 2018; Lee et al., 2020).

Female sex significantly influenced the likelihood of ST-T abnormalities - even after adjusting for HRV parameters. This finding aligns with prior research indicating sex-specific differences in autonomic regulation and cardiovascular risk profiles. Women have been reported to exhibit higher parasympathetic tone and HRV indices under resting conditions, but may also experience unique autonomic responses under stress or cardiac strain, potentially affecting ECG patterns differently than in men (Koenig and Thayer, 2016).

An increased cardiovascular risk associated with night-shift work has been reported previously (Morris et al., 2016; Cannizzaro et al., 2020). One possible explanation for reduced HRV during nocturnal shifts is decreased parasympathetic activity in individuals who are awake and active during this period (Jensen et al., 2016; Cheng et al., 2019). Differences in HRV between on- and off-duty states have also been documented. For instance, Schoeniger et al. reported significant differences in SDNN values—but not RMSSD—between rest periods during and outside of duty hours, though absolute differences were small (Schöniger et al., 2020a; 2020b).

Both ST-T segment abnormalities and reduced HRV are recognized as predictors of cardiovascular risk. The presence of each in the current sample underscores the elevated cardiac strain experienced by prehospital emergency physicians, despite the absence of a direct correlation between the two measures.

SDNN values in the present sample were generally low, with a mean below the 100 m threshold commonly regarded as indicative of cardiovascular health (Shaffer and Ginsberg, 2017). This is in contrast to data presented previously where SDNN values were above 100 m as it would be expected (Petrowski et al., 2019). One possible explanation could be the long-lasting effects of stress–even on days off duty. This has been described in the context of long term stress both in occupational and care-giving settings (Järvelin-Pasanen et al., 2018; da Estrela et al., 2021).

Other studies involving high-stress environments like surgery showed the same inconsistency as our results. While a study investigating the effects of 20-h surgical shifts did not find changes in time-domain HRV parameters, alterations in frequency-domain components indicated a shift in autonomic nervous system balance (Langelotz et al., 2008). In contrast, a study adding data from the State Trait Anxiety Inventory could show that stressed surgeons had decrease HRV values (Rieger et al., 2014).

Recording HRV in physicians was previously done not only in emergency physicians but also in surgeons: It was shown that stress persisted long after surgery and that individual psychological characteristics are more important than experience (Carnevali et al., 2023). Even the type of surgery influenced HRV metrics as parameters of stress (Böhm et al., 2001).

Internal consistency of the dataset is supported by expected HRV patterns across mission phases. Both SDNN and RMSSD values were significantly lower during patient care and transport—phases typically associated with heightened stress (Schöniger et al., 2020b). In contrast ST-T segment changes were most frequently observed during the alarm and patient care phases (Maleczek et al., 2022). This further emphasizes the inconsistency between HRV and ST-T segment changes. This could lead to the assumption that stress perceived during the alarm has different cardiovascular effects than the one during patient care.

Comparison of RMSSD and SDNN showed both values tending in the same direction, but SDNN showed greater absolute differences between groups than RMSSD. This can be mathematically explained as RMSSD has a smaller range of values due to calculation of a square root. Interestingly, for some outcomes, e.g., during time of immediate ST-T segment changes, SDNN and RMSSD values diverged in opposite directions.

### 4.1 Limitations

Despite the prospective nature of data collection, this analysis was retrospective and may be subject to selection and analytical bias. Additionally, reliance on automated software for HRV computation introduces a potential source of systematic error. However, this risk appears minimal, given the software’s demonstrated QRS detection accuracy (99.9% on MIT-BIH datasets, according to Schiller AG) (Moody and Mark, 2001). A point of particular importance is the treatment of atypical beats, such as those with altered ST-T segment changes. These beats were intentionally included in the analysis to allow for a direct comparison between ST-T segment changes and HRV. Only beats for which the software was unable to reliably detect the R wave were excluded.

As this trial was a preplanned secondary analysis, one has to keep in mind the possibly introduced bias. Therefore, results are hypothesis generating only.

Another important limitation of this trial is the 5-min interval used for both metrics: For both metrics different time intervals are described while external outcome driven validation is missing for most of those (DeGiorgio et al., 2010). Therefore, the compensation of short artefacts caused by movement and the short duration of ST-T segment changes had to be balanced and a 5-min interval was chosen. Future studies using higher-resolution or event-triggered HRV analysis, possibly integrating beat-to-beat dynamics, may offer more precise insights into the physiological precursors of transient myocardial repolarization abnormalities.

T-wave inversions, while frequently used as indicators of repolarization abnormalities, are clinically ambiguous and must be interpreted with caution. They can result from a variety of conditions, including myocardial ischemia, electrolyte imbalances, hyperventilation, or heightened sympathetic activity, all of which may occur in high-stress prehospital environments. In the absence of concurrent clinical symptoms (no participant reported chest pain), the presence of T-wave changes alone cannot reliably indicate pathology (Kligfield et al., 2007; Byrne et al., 2023). This inherent ambiguity limits the specificity of ST-T abnormalities as endpoints and underscores the need for careful contextualization when linking ST-T abnormalities to autonomic markers such as HRV.

Beyond the immediate clinical and psychological implications, our findings contain significant translational value. A key result of this study is that HRV alone is insufficient to capture the full extent of stress-related cardiac alterations. Instead, comprehensive cardiac monitoring using a 12-lead ECG in combination with HRV is necessary to detect changes associated with occupational stress. Interventions based on these findings—such as targeted stress-reduction programs, organizational changes to workload and scheduling - may not only improve physician wellbeing but also enhance patient safety and healthcare system resilience. Future studies should evaluate the implementation of these approaches with the goal of reducing occupational stress, improving long-term health outcomes, and potentially contributing to increased life expectancy among physicians.

## 5 Conclusion

In prehospital emergency physicians, higher SDNN values were associated with both a risk of immediate ST-T abnormalities and ST-T abnormalities on mission level. RMSSD was suitable to predict ST-T segment with a negative association during missions but not immediate ST-T abnormalities. All HRV metrics were higher in the alarm phase and en-route phase compared to patient care and patient transport.

## Data Availability

The raw data supporting the conclusions of this article will be made available by the authors, without undue reservation.
