# Supplementary material for: Association between heart rate variability and ECG changes in on-duty prehospital physicians
Source: Front Physiol. 2025 Jul 23;16:1617377. doi: 10.3389/fphys.2025.1617377 (PMC12325379; doi:10.3389/fphys.2025.1617377)
Supplement: Supplementary file 1 [file DataSheet1.docx]

# Suppplemental Material 1: Code

## R-Code

Python:

#mixed effect logistische regression mit lme4

df <- read.csv("XXXX")

library(janitor)

df <- janitor::clean_names(df)

df$zeit <- as.difftime(df$zeit)

library(lme4)

df$ecg_change <- as.factor(df$ecg_change)

library(janitor)

df <- clean_names(df)

str(df)

model <- glmer(

ecg_change ~ sdnn_ms + r_mssd_ms + p_nn50 + (1 | proband),

data = df,

family = binomial

)

summary(model)

# Calculate the Odds Ratios (OR)

odds_ratios <- exp(fixef(model))

# Calculate the 95% confidence intervals for the fixed effects using the "Wald" method

conf_ints <- confint(model, method = "Wald", level = 0.95)

# Exponentiate the confidence intervals to get the CI for odds ratios

conf_ints_OR <- exp(conf_ints)

# Create a data frame of Odds Ratios and Confidence Intervals

print(names(odds_ratios))

print(odds_ratios)

print(conf_ints_OR)

# Forest Plot

library(broom.mixed)

library(ggplot2)

library(dplyr)

# Tidy the model output

tidy_model <- tidy(model, effects = "fixed", conf.int = TRUE, exponentiate = TRUE)

# Rename terms for clarity

tidy_model <- tidy_model %>%

mutate(term = recode(term,

"sdnn_ms" = "SDNN",

"r_mssd_ms" = "RMSSD",

"p_nn50" = "pNN50"

))

# Filter and order terms for plotting

tidy_model <- tidy_model %>%

filter(term != "(Intercept)") %>%

arrange(desc(estimate)) %>%

mutate(term = factor(term, levels = term)) # Keep order in plot

# Highlight significant effects

tidy_model <- tidy_model %>%

mutate(Significance = ifelse(conf.low > 1 | conf.high < 1, "Significant", "Not significant"))

# Forest plot

ggplot(tidy_model, aes(x = estimate, y = term, color = Significance)) +

geom_point(size = 3) +

geom_errorbarh(aes(xmin = conf.low, xmax = conf.high), height = 0.2, linewidth = 1) +

geom_vline(xintercept = 1, linetype = "dashed", color = "grey40") +

scale_x_log10() + # Optional: use log scale for better visualization of odds ratios

scale_color_manual(values = c("Significant" = "#D55E00", "Not significant" = "grey40")) +

labs(

title = "Association between HRV Parameters and ECG Changes",

x = "Odds Ratio (log scale, 95% CI)",

y = "HRV Metric"

) +

theme_minimal(base_size = 14) +

theme(

axis.text.y = element_text(size = 13),

axis.title = element_text(size = 14),

plot.title = element_text(size = 16, face = "bold", hjust = 0.5),

legend.title = element_blank(),

legend.position = "bottom"

)

# on mission level

df2 <- read.csv("XXX")

str(df2)

library(janitor)

df2 <- clean_names(df2)

str(df2)

library(lme4)

model2 <- glmer(

bin_ecg ~ sdnn_overall_mean + pnn50_overall_mean + rmssd_overall_mean + (1 | prob_id),

data = df2,

family = binomial

)

summary(model2)

# Calculate the Odds Ratios (OR)

odds_ratios <- exp(fixef(model2))

# Calculate the 95% confidence intervals for the fixed effects using the "Wald" method

conf_ints <- confint(model2, method = "Wald", level = 0.95)

# Exponentiate the confidence intervals to get the CI for odds ratios

conf_ints_OR <- exp(conf_ints)

# Ensure that the odds_ratios and the confidence intervals align

# Create a data frame of Odds Ratios and Confidence Intervals

print(names(odds_ratios))

print(odds_ratios)

print(conf_ints_OR)

## Forenst plot

library(broom.mixed)

library(ggplot2)

library(dplyr)

# Tidy the model output (fixed effects only)

tidy_model2 <- tidy(model2, effects = "fixed", conf.int = TRUE, exponentiate = TRUE)

# Rename HRV variables for readability

tidy_model2 <- tidy_model2 %>%

mutate(term = recode(term,

"sdnn_overall_mean" = "SDNN",

"rmssd_overall_mean" = "RMSSD",

"pnn50_overall_mean" = "pNN50"

))

# Filter, order, and format

tidy_model2 <- tidy_model2 %>%

filter(term != "(Intercept)") %>%

arrange(desc(estimate)) %>%

mutate(term = factor(term, levels = term),

Significance = ifelse(conf.low > 1 | conf.high < 1, "Significant", "Not significant"))

# Create forest plot

ggplot(tidy_model2, aes(x = estimate, y = term, color = Significance)) +

geom_point(size = 3) +

geom_errorbarh(aes(xmin = conf.low, xmax = conf.high), height = 0.2, linewidth = 1) +

geom_vline(xintercept = 1, linetype = "dashed", color = "grey40") +

scale_x_log10() + # Log scale for odds ratio

scale_color_manual(values = c("Significant" = "#D55E00", "Not significant" = "grey40")) +

labs(

title = "Association Between HRV Metrics and ECG Changes per Mission",

x = "Odds Ratio (log scale, 95% CI)",

y = "HRV Metric"

) +

theme_minimal(base_size = 14) +

theme(

axis.text.y = element_text(size = 13),

axis.title = element_text(size = 14),

plot.title = element_text(size = 16, face = "bold", hjust = 0.5),

legend.title = element_blank(),

legend.position = "bottom"

)

## supplement with sex:

df <- read.csv("XXX")

library(janitor)

df <- janitor::clean_names(df)

df$zeit <- as.difftime(df$zeit)

library(lme4)

df$ecg_change <- as.factor(df$ecg_change)

df <- clean_names(df)

str(df)

model <- glmer(

ecg_change ~ sdnn_ms + r_mssd_ms + p_nn50 + sex + (1 | proband),

data = df,

family = binomial

)

summary(model)

# Calculate the Odds Ratios (OR)

odds_ratios <- exp(fixef(model))

# Calculate the 95% confidence intervals for the fixed effects using the "Wald" method

conf_ints <- confint(model, method = "Wald", level = 0.95)

# Exponentiate the confidence intervals to get the CI for odds ratios

conf_ints_OR <- exp(conf_ints)

# Create a data frame of Odds Ratios and Confidence Intervals

print(names(odds_ratios))

print(odds_ratios)

print(conf_ints_OR)

## supplement with heart rate:

df <- read.csv("XXX")

library(janitor)

df <- janitor::clean_names(df)

df$zeit <- as.difftime(df$zeit)

library(lme4)

df$ecg_change <- as.factor(df$ecg_change)

df <- clean_names(df)

str(df)

library(lme4)

model <- glmer(

ecg_change ~ sdnn_ms + r_mssd_ms + p_nn50 + mittel_bpm + (1 | proband),

data = df,

family = binomial

)

summary(model)

# Calculate the Odds Ratios (OR)

odds_ratios <- exp(fixef(model))

# Calculate the 95% confidence intervals for the fixed effects using the "Wald" method

conf_ints <- confint(model, method = "Wald", level = 0.95)

# Exponentiate the confidence intervals to get the CI for odds ratios

conf_ints_OR <- exp(conf_ints)

# Ensure that the odds_ratios and the confidence intervals align

# Create a data frame of Odds Ratios and Confidence Intervals

print(names(odds_ratios))

print(odds_ratios)

print(conf_ints_OR)

## Python

from itertools import combinations

from scipy.stats import ttest_rel

def run_paired_ttests(df, metrics, phases, alpha=0.05):

phase_label_map = {

'alarm': 'Alarm',

'zBO': 'En-route',

'pat_care': 'Patient Care',

'Transport': 'Transport'

}

results = []

for metric in metrics:

for phase1, phase2 in combinations(phases, 2):

col1 = f'{metric}_{phase1}_mean'

col2 = f'{metric}_{phase2}_mean'

# Drop rows with missing values for either column

valid_data = df[[col1, col2]].dropna()

# Paired t-test

t_stat, p_value = ttest_rel(valid_data[col1], valid_data[col2])

results.append({

'Metric': metric.upper(),

'Phase 1': phase_label_map[phase1],

'Phase 2': phase_label_map[phase2],

't-statistic': t_stat,

'p-value': p_value

})

# Adjust p-values using Bonferroni correction

result_df = pd.DataFrame(results)

result_df['p-value (Bonferroni)'] = result_df['p-value'] * len(result_df)

result_df['Significant'] = result_df['p-value (Bonferroni)'] < alpha

return result_df

# Define metrics and phases

metrics = ['rmssd', 'sdnn', 'pnn50']

phases = ['alarm', 'zBO', 'pat_care', 'Transport']

# Run paired t-tests

ttest_results = run_paired_ttests(crf_hrv, metrics, phases)

# Display results

print(ttest_results)

def get_significant_pairs(df, metrics, phases, alpha=0.05):

from itertools import combinations

from scipy.stats import ttest_rel

phase_label_map = {

'alarm': 'Alarm',

'zBO': 'En-route',

'pat_care': 'Patient Care',

'Transport': 'Transport'

}

results = {}

for metric in metrics:

sig_pairs = []

all_tests = []

for phase1, phase2 in combinations(phases, 2):

col1 = f'{metric}_{phase1}_mean'

col2 = f'{metric}_{phase2}_mean'

valid = df[[col1, col2]].dropna()

t_stat, p_val = ttest_rel(valid[col1], valid[col2])

all_tests.append((phase1, phase2, p_val))

# Bonferroni correction

corrected = [(a, b, p, p * len(all_tests)) for a, b, p in all_tests]

sig_pairs = [(a, b, p_corr) for a, b, p, p_corr in corrected if p_corr < alpha]

results[metric] = sig_pairs

return results

import matplotlib.pyplot as plt

import seaborn as sns

import pandas as pd

def plot_merged_boxplots_with_significance_pub(df, metrics, phases, sig_results, save_path = None):

phase_label_map = {

'alarm': 'Alarm',

'zBO': 'En-route',

'pat_care': 'Patient Care',

'Transport': 'Transport'

}

metric_label_map = {

'rmssd': 'RMSSD [ms]',

'sdnn': 'SDNN [ms]',

'pnn50': 'pNN50'

}

sns.set(style='whitegrid', context='notebook', font_scale=1.2)

plt.figure(figsize=(12, 8))

for i, metric in enumerate(metrics, start=1):

metric_cols = [f'{metric}_{phase}_mean' for phase in phases]

metric_data = df[metric_cols].copy()

# Rename columns to phase labels for plotting

metric_data.columns = [phase_label_map[p] for p in phases]

melted = metric_data.melt(var_name='Phase', value_name='Value')

ax = plt.subplot(1, len(metrics), i)

sns.boxplot(

x='Phase', y='Value', data=melted, palette="Set2",

width=0.6, linewidth=1.5, ax=ax, fliersize=3

)

#ax.set_ylim(bottom=0)

# Set x-axis tick labels explicitly

ax.set_xticklabels(list(phase_label_map.values()), rotation=45, fontsize=12)

# Titles and labels

ax.set_title(metric_label_map[metric], fontsize=16, weight='bold')

ax.set_xlabel('Mission Phase', fontsize=14)

ax.set_ylabel(metric_label_map[metric], fontsize=14)

ax.tick_params(labelsize=12)

# Annotate significance

sig_pairs = sig_results.get(metric, [])

phase_labels = [phase_label_map[p] for p in phases]

y_max = melted['Value'].max()

y_min = melted['Value'].min()

spacing = (y_max - y_min) * 0.05

for idx, (p1, p2, p_corr) in enumerate(sig_pairs):

x1 = phase_labels.index(phase_label_map[p1])

x2 = phase_labels.index(phase_label_map[p2])

y = y_max + spacing * (idx + 1)

stars = '***' if p_corr < 0.001 else '**' if p_corr < 0.01 else '*'

ax.plot([x1, x1, x2, x2], [y, y + spacing * 0.2, y + spacing * 0.2, y], lw=1.5, color='black')

ax.text((x1 + x2) / 2, y + spacing * 0.25, stars,

ha='center', va='bottom', fontsize=14, weight='bold')

sns.despine(trim=True)

ax.grid(axis='y', linestyle='--', linewidth=0.5)

if save_path:

plt.savefig(save_path, dpi=300, bbox_inches='tight') # Save high-res figure with tight layout

plt.tight_layout()

plt.show()

# Run paired t-tests

sig_results = get_significant_pairs(crf_hrv, metrics, phases)

# Plot boxplots with significance annotations

plot_merged_boxplots_with_significance_pub(crf_hrv, metrics, phases, sig_results, save_path='Figure 3_1.png')
